# Pelvic Diagnostic Challenges of Appendiceal Neoplasm Mimicking a Hematosalpinx: A Case Report

**DOI:** 10.7759/cureus.61945

**Published:** 2024-06-08

**Authors:** Amro Bannan, Ali Mourad, Bich N Nguyen, Roland Antaki

**Affiliations:** 1 Obstetrics and Gynaecology, University of Jeddah, Jeddah, SAU; 2 Obstetrics and Gynaecology, University of Montreal Health Centre (CHUM), Montreal, CAN; 3 Pathology and Laboratory Medicine, University of Montreal Health Centre (CHUM), Montreal, CAN; 4 Obstetrics and Gynaecology, Ovo Fertility Center, Montreal, CAN

**Keywords:** fertility preservation, infertility, pseudomyxoma peritonei, low-grade appendiceal mucinous neoplasm, primary infertility, appendiceal mucinous neoplasm, adnexal masses, appendiceal neoplasm mimicking a hematosalpinx, hematosalpinx

## Abstract

Appendiceal mucinous neoplasms are rare and can be easily misdiagnosed as adnexal masses. Fertility is a concern in cases requiring cytoreductive surgery involving the ovaries and if hyperthermic intraperitoneal chemotherapy is considered. We present the case of a 35-year-old patient with primary infertility who was suspected to have a hematosalpinx on ultrasonography and magnetic resonance imaging (MRI) but was found to have an appendiceal mucinous neoplasm on laparoscopy. Fertility preservation was offered to this patient. Appendiceal mucinous neoplasms should be considered in the differential diagnosis of patients in their reproductive years presenting with adnexal masses. Fertility preservation should be discussed with these patients, especially when gonadotoxic treatments are planned.

## Introduction

Appendiceal mucinous neoplasms (AMNs) are a rare pathological entity, often presenting as incidental findings during radiologic evaluations or endoscopic/operative procedures [1]. The age-adjusted incidence of AMNs is 0.12 cases per one million individuals per year, with a slight female predominance [2]. Multiple case reports have shown that AMNs can be easily misdiagnosed as adnexal masses [3,4]. Adnexal masses of the ovary, fallopian tube, or surrounding tissue are common in females of all ages, with a prevalence of 7.8% on ultrasonography in asymptomatic women during their reproductive years [5]. Therefore, AMNs should be considered in the differential diagnosis of adnexal masses, though infrequent.

The classic diagnostic evaluation of adnexal masses involves imaging studies (mainly ultrasonography, magnetic resonance imaging (MRI), or computed tomography (CT)) and laboratory studies (tumor markers including CEA, CA19-9, and CA-125) [6]. Despite similar diagnostic tools for the evaluation of AMNs, these pathologies are often misdiagnosed [4]. Multiple case reports describe the diagnosis of AMNs during pregnancy or a cesarean section [7]; however, infertility is one of the possible unusual presentations. Fertility preservation is a significant concern for women diagnosed with AMNs or pseudomyxoma peritonei requiring cytoreductive surgeries that might include the ovaries or treatment using hyperthermic intraperitoneal chemotherapy (HIPEC) [8]. We present the case of a low-grade appendiceal mucinous neoplasm (LAMN) diagnosed during an infertility evaluation.

## Case presentation

**Figure 1:**
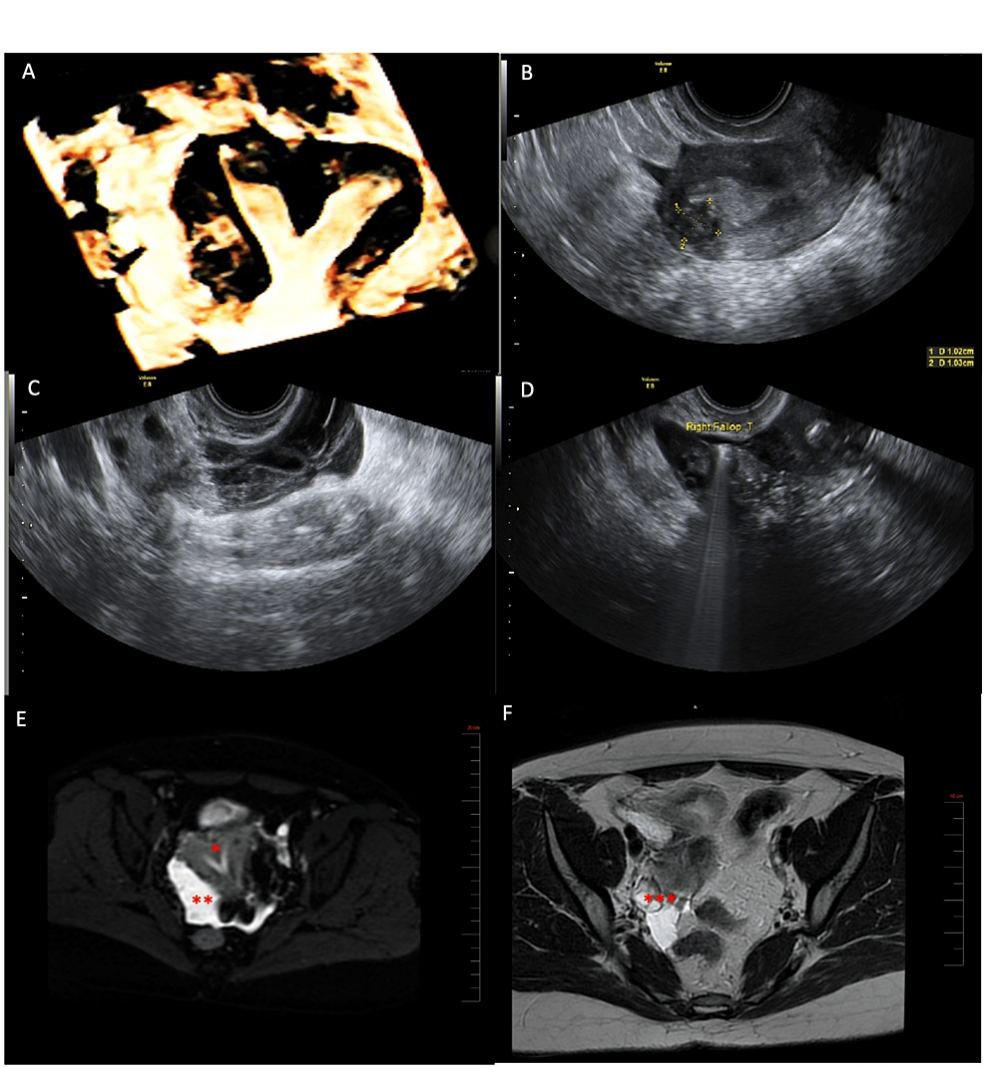
(A) 3D ultrasound of the uterus showing the uterine septum and the type I fibroid. (B) 2D ultrasound of the uterus showing the uterine fibroid. (C) Heterogeneous fluid next to the right ovary mimicking a hematosalpinx. (D) Patent right fallopian tube on sonohysterosalpingography. (E) MRI showing septated uterus (*) and suspicion of right-sided hydrosalpinx (**). (F) MRI showing the right ovary (***) surrounded by fluid accumulation. MRI: magnetic resonance imaging

A 35-year-old woman presented to our clinic with primary infertility for 18 months. She had a negative medical, surgical, and family history. Transvaginal ultrasonographic evaluation showed a 2 cm uterine septum and a 1x1 cm type I fibroid (according to the International Federation of Gynaecology and Obstetrics (FIGO) classification) (Figure 1A, 1B). Additionally, heterogeneous fluid accumulation next to the right ovary was detected, raising the suspicion of a hematosalpinx (Figure 1C). However, a sonohysterosalpingography showed a normal patent right fallopian tube (Figure 1D). An MRI, along with tumor markers, was subsequently ordered. The MRI confirmed the uterine septum and showed right tubal dilation up to 1 cm with a minimal amount of free fluid in the posterior cul-de-sac (Figure 1E, 1F). Tumor markers were within the normal range: CA19-9 8 kU/L, CEA 2.9 µg/L, and CA-125 17 kU/L.

**Figure 2:**
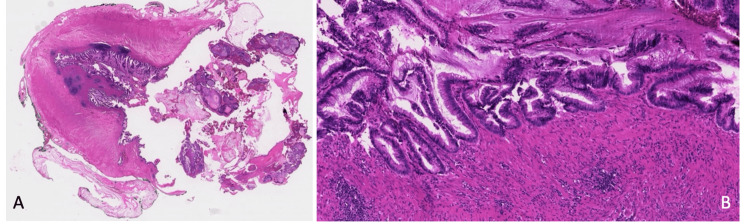
(A) Low-grade mucinous neoplasm with a "pushing" pattern of growth resulting in the rupture of the appendiceal wall and acellular mucinous deposits on the visceral peritoneal surface (and diffuse peritoneal dissemination, not shown) (H&E, ×5). (B) The neoplastic mucinous epithelium has a villous architectural arrangement and low-grade dysplasia (H&E, ×100).

A decision was made to perform a diagnostic laparoscopy with chromotubation, and the patient consented to a possible right salpingectomy. The laparoscopy revealed mucin in the posterior cul-de-sac with mild adhesions between the right ovary and the pelvic wall. Mucin aspiration, pelvic adhesiolysis, and a peritoneal biopsy were performed. The fluid was sent for cytology. Examination of the appendix revealed a perforated AMN, and an appendectomy was performed. The specimen was removed with an endobag and sent for pathology without contamination. A complete exploration of the abdominal cavity did not detect any other lesions. A hysteroscopic septoplasty and myomectomy were also performed. Pathology confirmed the presence of a LAMN with diffuse acellular mucin (pathologic stage pT4M1a) (Figure 2A, 2B). In case of recurrence, a laparoscopy followed by possible HIPEC was discussed with the patient. A follow-up CT scan was repeated six months postoperatively, showing no recurrence of the disease. Fertility preservation was offered, and an in vitro fertilization (IVF) antagonist cycle was conducted in November 2021 with 450 IU of gonadotropins. A total of 23 eggs were retrieved, 20 of which were mature. Intracytoplasmic sperm injection (ICSI) was performed, resulting in the fertilization of 15 eggs and the formation of eight blastocysts, which were subsequently frozen. Following consultations with oncology and surgery, the patient was cleared for pregnancy. A medicated frozen embryo transfer with hormone replacement of a single blastocyst was successful 10 months after the initial surgery, and the patient had an uneventful pregnancy with no complications, delivering vaginally a healthy live birth at term. She still has seven embryos frozen.

## Discussion

Mucocele of the appendix is characterized by abnormal mucus accumulation, leading to appendix distension and presenting significant diagnostic challenges due to its mimicry of other abdominal or pelvic pathologies. The clinical presentation is highly variable, with common symptoms including right lower quadrant pain, abdominal pain, and distension, which often lead to preoperative diagnoses such as ovarian cysts, pelvic inflammatory disease, and tubo-ovarian abscesses. For instance, in the case reported by Bahia and Wilson [9], the patient presented with a right adnexal mass initially suspected to be a hydrosalpinx or tubo-ovarian abscess, while Kalu and Croucher [10] reported an incidental finding of an ovarian cyst. The variability in clinical presentation, coupled with overlapping symptoms with other conditions, complicates accurate diagnosis. Imaging modalities like ultrasound, CT, and MRI are crucial in identifying mucocele; however, these can sometimes lead to misdiagnosis, as seen in cases by Abu Zidan et al. [11] and Akman et al. [12], where twisted ovarian cysts and ovarian torsion were initially suspected.

The differential diagnosis is further complicated by the potential for malignant transformation, necessitating a high index of suspicion to guide appropriate surgical management. The cases by Suh et al. [13] and Panagopoulos et al. [14] illustrate the importance of detailed imaging and careful surgical planning. A review by Tirumani et al. [15] emphasizes the role of imaging in identifying the diverse presentations of mucinous neoplasms, highlighting the importance of recognizing extra-appendiceal mucin as a critical factor in staging and prognosis. Shaib et al. [16] discuss the heterogeneous nature of AMNs and the challenges in their classification and treatment. This review underscores the need for awareness and consideration of appendiceal mucocele in cases of right lower quadrant pain and adnexal masses. By consolidating knowledge from various case reports and series, it highlights the importance of considering this rare entity in differential diagnosis to improve diagnostic accuracy and patient outcomes.

We have also reported a rare case of LAMN diagnosed during an infertility evaluation. Limited cases addressing the misdiagnosis of gastrointestinal pathologies have been reported, highlighting the complexity and pitfalls in diagnosing pelvic masses. The most frequently reported cases involve appendiceal pathologies, specifically mucinous adenocarcinoma [17]. Our report is the first to address fertility in the context of a misdiagnosed LAMN. Fertility preservation should be discussed with patients during their reproductive years before HIPEC or cytoreductive surgeries involving the ovaries.

The appendix is currently a topic of interest in gynecologic surgeries, mainly in the management of endometriosis and dyspareunia. Limited evidence suggests a benefit from concomitant appendectomy in endometriosis, even in cases of morphologically normal appendices [18]. While this should not be generalized for all diagnostic or gynecologic procedures, inspection of the appendix is a good practice point that should be integrated into all such surgeries.

Finally, fertility preservation is best proposed before any treatments that might affect fertility in reproductive-age women [19]. It is the clinician's duty to provide information about fertility preservation options to these patients. Unfortunately, referral rates for fertility preservation are still consistently low [20], highlighting the need for increased awareness among clinicians from different specialties.

## Conclusions

This case report and literature review highlights the diagnostic challenges posed by mucocele of the appendix due to its varied presentation and overlap with other abdominal and pelvic pathologies. The findings underscore the importance of considering this rare entity in the differential diagnosis of adnexal masses.
